# Microarray analysis of relative gene expression stability for selection of internal reference genes in the rhesus macaque brain

**DOI:** 10.1186/1471-2199-11-47

**Published:** 2010-06-21

**Authors:** Nigel C Noriega, Steven G Kohama, Henryk F Urbanski

**Affiliations:** 1 Division of Neuroscience, Oregon National Primate Research Center, 505 NW 185th Avenue, Beaverton, OR 97006, USA; 2 Department of Behavioral Neuroscience, Oregon Health and Science University, 3181 SW Sam Jackson Park Road, Portland, OR 97239, USA; 3 Department of Physiology and Pharmacology, Oregon Health and Science University, 3181 SW Sam Jackson Park Road, Portland, OR 97239, USA; 4 Department of Neurobiology Physiology and Behavior, Center for Neuroscience 1273, University of California, Davis, CA 95618, USA

## Abstract

**Background:**

Normalization of gene expression data refers to the comparison of expression values using reference standards that are consistent across all conditions of an experiment. In PCR studies, genes designated as "housekeeping genes" have been used as internal reference genes under the assumption that their expression is stable and independent of experimental conditions. However, verification of this assumption is rarely performed. Here we assess the use of gene microarray analysis to facilitate selection of internal reference sequences with higher expression stability across experimental conditions than can be expected using traditional selection methods.

We recently demonstrated that relative gene expression from qRT-PCR data normalized using *GAPDH*, *ALG9 *and *RPL13A *expression values mirrored relative expression using quantile normalization in Robust Multichip Analysis (RMA) on the Affymetrix^® ^GeneChip^® ^rhesus Macaque Genome Array.

Having shown that qRT-PCR and Affymetrix^® ^GeneChip^® ^data from the same hormone replacement therapy (HRT) study yielded concordant results, we used quantile-normalized gene microarray data to identify the most stably expressed among probe sets for prospective internal reference genes across three brain regions from the HRT study and an additional study of normally menstruating rhesus macaques (cycle study). Gene selection was limited to 575 previously published human "housekeeping" genes. Twelve animals were used per study, and three brain regions were analyzed from each animal. Gene expression stabilities were determined using geNorm, NormFinder and BestKeeper software packages.

**Results:**

Sequences co-annotated for ribosomal protein S27a (*RPS27A*), and ubiquitin were among the most stably expressed under all conditions and selection criteria used for both studies. Higher annotation quality on the human GeneChip^® ^facilitated more targeted analysis than could be accomplished using the rhesus GeneChip^®^. In the cycle study, multiple probe sets annotated for actin, gamma 1 (*ACTG1*) showed high signal intensity and were among the most stably expressed.

**Conclusions:**

Using gene microarray analysis, we identified genes showing high expression stability under various sex-steroid environments in different regions of the rhesus macaque brain. Use of quantile-normalized microarray gene expression values represents an improvement over traditional methods of selecting internal reference genes for PCR analysis.

## Background

Quantitative real-time reverse transcription polymerase chain reaction (qRT-PCR) [1,2] has been the technique of choice to confirm or refute interpretations of relative gene expression derived from gene microarray data [3-6], but accurate interpretation requires the qRT-PCR gene expression levels to be normalized against a stably expressed reference [7]. Genes such as Glyceraldehyde-3- phosphate dehydrogenase (GAPDH), β-actin (*ACTB*) and 18S rRNA have been used as internal reference standards for qRT-PCR normalization mainly due to historical carryover, when they were used as references for semi-quantitative procedures like Northern blots, RNAse protection assays and conventional reverse transcriptase polymerase chain reactions (RT-PCR) [8]. However expression of these genes is regulated according to cellular conditions [9-15], and so their indiscriminate use as internal reference genes is questionable. Many genes previously adopted as standards for normalization of gene expression data show variable expression according experimental conditions [11,15], thus limiting their suitability. Increasingly, it is viewed as imperative that the expression stability of prospective reference genes be verified under each all experimental conditions specific to a study [16-18]. Gene expression analysis using microarrays facilitates use of an increasingly more complete range of sample variation criteria [19], and studies using gene microarray analyses are an increasingly common means of identifying experiment-specific internal reference genes for use in PCR verification [20-24].

Few suitable reference genes have been identified within the rhesus macaque brain [25]. Normalizer data for macaque brain tissues is sparse because the availability of genetic material from specific brain regions in properly controlled groups of macaques is very rare and difficult to obtain. To help overcome this problem, we recently used qRT-PCR and gene microarrays to examine expression stability for genes regulating γ-aminobutyric acid (GABA) trafficking in response to variation in circulating levels of ovarian steroids in the arcuate nucleus of the medial basal hypothalamus (MBH), hippocampus (HPC) and amygdala (AMD) in a study designed for examination of effects of hormone replacement therapy on gene expression (HRT study). In the aforementioned analysis [26], we showed that qRT-PCR results normalized using genes showing high microarray expression stability (*GAPDH*, *ALG9 *and *RPL13A*) mirrored relative microarray expression data normalized using the quantile method [27,28] as part of Robust Multichip Analysis (RMA) pre-processing [29] on the Affymetrix^® ^GeneChip^® ^rhesus Macaque Genome Array (rhesus GeneChip^®^) [26].

We now present further details of our qRT-PCR relative expression stability analyses using the software algorithm bundles: geNorm [30], NormFinder [31] and BestKeeper [32]. We compare these data with relative gene expression stabilities from quantile-normalized RMA pre-processed (RMA-normalized) microarray results from the same (HRT) study examined using the rhesus GeneChip^®^. In addition we compare relative expression stabilities from a separate study of normally cycling macaques (cycle study) where we conducted microarray analysis of the same brain regions (MBH, HPC and AMD) using the Affymetrix^® ^GeneChip^® ^Human Genome U133 Plus 2.0 microarray (human GeneChip^®^).

In order to maximize the likelihood of detecting gene sequences that are stably expressed under a variety of conditions (i.e., different brain regions and different sex-steroid environments), gene selection was limited to a published set of 575 human genes expressed under all conditions tested [33], and expected to meet criteria for "housekeeping" genes. Our goal was to identify the most appropriate normalizer or combination of normalizers for gene expression comparisons, and we selected these criteria to limit inclusion of probe sets to those most likely to be good candidates under variable conditions [22,34]. The experiments conducted in the current study were designed to test effects of variable ovarian hormone exposure on the function of three brain areas, and we used menstrual cycle variation and hormone therapy effects as two ways of independently assessing ovarian hormone effects.

## Results

**Figure 1:**
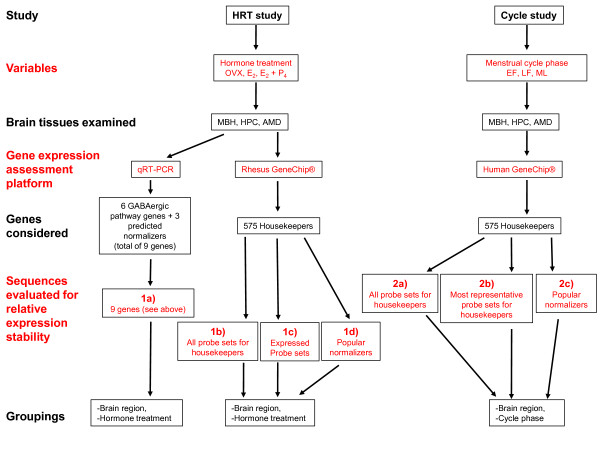
**Study methodology schematic**. Flowchart outlining organization and groupings for analyses of data from the HRT and cycle studies. Alternating black and red text color is used for association of headings (left column) with appropriate rows of flowchart boxes. The six GABAergic pathway genes and three predicted normalizers for qRT-PCR are detailed in the methods. Numbered designations (1a, 1b... 2c) refer to separate selections of sequences to be analyzed (detailed in methods), with HRT sequences from 1a to 1c, and cycle study sequences from 2a to 2c. AMD = amygdala; E_2 _= 17β-estradiol; E_2_+P_4 _= 17β-estradiol + progesterone; EF = early follicular (menstrual cycle phase); GABA = gamma-aminobutyric acid; HPC = hippocampus; LF = late follicular (menstrual cycle phase); MBH = arcuate nucleus of the medial basal hypothalamus; ML = mid luteal (menstrual cycle phase); OVX = ovariectomized; qRT-PCR = quantitative real-time polymerase chain reaction;

The results were obtained from two different experiments, which are described in the methods and depicted schematically in Figure 1. Previously qRT-PCR was conducted on samples from the HRT study in order to examine the influence of hormone replacement on gene expression of GABAergic system components [26]. qRT-PCR relative expression stabilities of the six GABAergic components and three normalizers used in the prior qRT-PCR analysis were assessed in the current study, and we compare analyses for these nine genes (Table 1) used for qRT-PCR to analyses of 575 housekeeping genes examined using gene microarray data pre-processed using Robust Multiarray Analysis (RMA normalization) [27,35,36]. Differences in annotation quality between the rhesus and human microarray platforms facilitated higher discrimination in probe set selection using the human compared to the rhesus GeneChip^®^. Because the nature of the probe sets used can affect overall relative expression stability values [37], we conducted separate analyses according to annotation-based on probe set selection criteria and present the gene microarray results according these selection methods. On both the human and rhesus GeneChip^® ^we compared: 1) all probe sets annotated for the housekeeping genes; and 2) probe sets annotated for "popular normalizers" (see methods). In addition to analysis of all probe sets annotated for housekeeping genes, the annotation quality of the human GeneChip^® ^facilitated selection of "most representative" probe sets (see methods) for the majority of the housekeeping genes as well as all of the popular normalizers. Use of the representative probe sets was intended to increase the specificity of data used for analyses.

**Table 1:** Primer and TAMRA probe sequence information for qRT-PCR

Gene name	Primer & probe sequences (5' to 3')		Reference sequence	Amplicon length
	F-primer	AACAGTGCCACAGAGCGAGAA		
*ALG9*	R-primer	CGATACCGCCTGGAGCACTA	XM_001106042	100
	probe	ACTGTCTTCCTGTTCGGG		
				
	F-primer	AAGGGCATCCTGGGCTACA		
*GAPDH*	R-primer	GAAGAGTGGGTGTCGCTGTTG	XM_001105471	66
	probe	TGAGCACCAGTGGTCTCCTCCGACT		
				
	F-primer	TCACGAGGTTGGCTGGAAGT		
*RPL13A*	R-primer	GATCTTGGCTTTCTCCTTCCTCTT	XM_001115079	72
	probe	CCAGGCAGTGACAGCCACCTTGG		
				
	F-primer	GGAAAATACACCGTGTTTTCCTAAA		
*GABRA1*	R-primer	AGGCAGGACCAAATCAAACAA	BV208518	72
	probe	ACACCTTTCTTTTTACATGTGCTTC		
				
	F-primer	TCCCTCTCTGCGTGTTTCAA		
*GABRD*	R-primer	GCCGAGGCTTCCTCTTGTTT	MMUGDNA.30046.1.S1_AT *	63
	probe	TGGGATGACAGTCGGCCACGG		
				
	F-primer	GGCACTGGAACTTTGGCAAA		
*GABRE*	R-primer	TGTCACAGGGCTATCATGAAGCT	BV166270	70
	probe	CACCTTTGACAAATTGTGTCTATTTG		
				
	F-primer	CTCCTCAACTATGTCCGCAAGA		
*GAD1*	R-primer	TCCAAGTTGAAGCCCTCCAT	NM_013445	100
	probe	TTCCATCACCCACACCAGTTGCTGG		
				
	F-primer	CTCGAAAGGCTTCAATTGCAT		
*GABRA4*	R-primer	CTTCCCAGTAGCCCCTATGGT	NM_000809	100
	probe	TGCAATCTTGATCCAAACACGTGACGA		
				
	F-primer	CACCATTGCCCGGAAATC		
*GABRG2*	R-primer	ACTCCACCAAAGCAGAGAAGACA	NM_198904	100
	probe	TCACAGCGATGGATCTCTTTGTATC		

F = forward, R = reverse. Reference sequence = NCBI accession except for *GABRD*.

* = Probe set sequence using NetAffx annotation for the GeneChip^® ^rhesus Macaque Genome Array http://www.affymetrix.com/analysis/index.affx

### qRT-PCR. HRT GeNorm analysis

**Figure 2:**
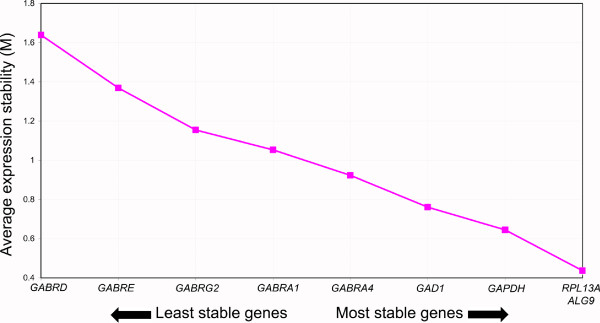
**Relative gene expression stability in the rhesus macaque brain**. GeNorm graphical output showing relative stability of genes expressed in three brain areas from three hormone replacement regimes. Stability values (M) are based on pairwise comparisons between the gene of choice and all other genes under consideration. The chart shows final stability values resulting from sequential elimination of the least stable genes after each set of pairwise analyses [34]. Therefore, the ordinate value shown for each gene represents the M-value obtained from the iteration before the gene was eliminated from analysis. Elimination sequence is shown from left to right. More stably expressed genes have lower M scores. Here the sample responsible for greatest influence on MBH variation was omitted from the analysis. When this sample was included the rank order of *GAPDH *and *GAD1 *were reversed.

Five of the original set of nine genes preselected for analysis showed initial expression stability (M) values [30] within the recommended range (M < 1.5) for consideration as a normalizer [34]. Where lower M indicates higher expression stability, initial M-value ranks (data not shown) were as follows: *ALG9 *<*GAPDH *<*RPL13A *<*GAD1 *<*GABRA4*. Sequential stepwise exclusion of the least stable reference genes resulted in final M-values of 0.437 for *ALG9 *and *RPL13A*, indicating that either of these genes was equally suitable for selection as the most stably expressed gene (best normalizer) according to the geNorm algorithm. Due to high variance introduced by a single MBH sample, it was removed and reanalysis was performed. The only effect of this change on relative M-values was regarding relative ranks of *GAD1 *and *GAPDH *with *GAPDH *showing higher stability after variation reduction (Figure 2). *ALG9 *and *RPL13A *remained the most stably expressed genes.

### qRT-PCR. HRT NormFinder analysis

*ALG9 *showed the highest stability when all three brain regions were compared. When all nine genes were analyzed, the five most stably expressed ones were identically ranked whether or not they were grouped [37] by brain region. The NormFinder algorithms work most efficiently when all genes used show high expression stability. From these nine genes, the five most stably expressed ones were selected. NormFinder algorithms were then applied to these five genes in order to narrow down the three most stably expressed. Because use of stably expressed genes is important for NormFinder function, we used this sequential method to ensure selection of genes with expression patterns that maximized the effectiveness of the NormFinder algorithm [37]. Where the top five gene candidates were considered separately, *ALG9 *was still the most stably expressed (stability value 0.255) and the most stable combination (stability value 0.150) was *GAPDH *+ *RPL13A *(Table 2). Where only the three prospective internal reference genes were considered, *ALG9 *was again the most stably expressed (0.204), and the most stable combination (0.152) was *GAPDH *and *ALG9*.

**Table 2:** NormFinder qRT-PCR expression stability analysis summary

Selection	MBH + HPC + AMD		MBH		HPC + AMD	
	Optimal single gene	Optimal gene combo	Optimal single gene	Optimal gene combo	Optimal single gene	Optimal gene combo
**From the five most stably expressed genes^§^**	*GAPDH*					*GAPDH*
	*ALG9*	+	*ALG9*	N/A	*GAPDH*	+
	0.255	*RPL13A*	0.120		0.037	*RPL13A*
	0.150					0.079
						
From the three most stably expressed genes*	*GAPDH*					*GAPDH*
	*ALG9*	+	*ALG9*	N/A	*GAPDH*	+
	0.204	*ALG9*	0.120		0.092	*ALG9*
		0.152				0.042

§ = *ALG9*, *RPL13A*, *GAPDH*, *GABRA4 *and *GABRA1*. * = *ALG9*, *RPL13A *and *GAPDH*

**Figure 3:**
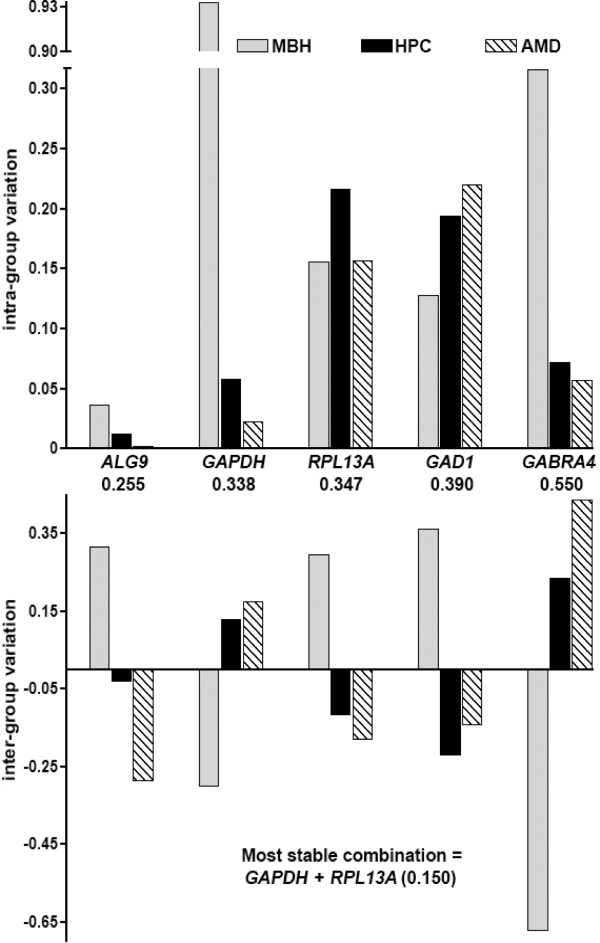
**NormFinder analysis for the five most stably expressed genes (qRT-PCR)**. Graphs show intra-group and inter-group variation [37] where gene expression quantity values were grouped according to brain region. Non-ordinate numbers show stability values for the given gene or gene combination.

**Figure 4:**
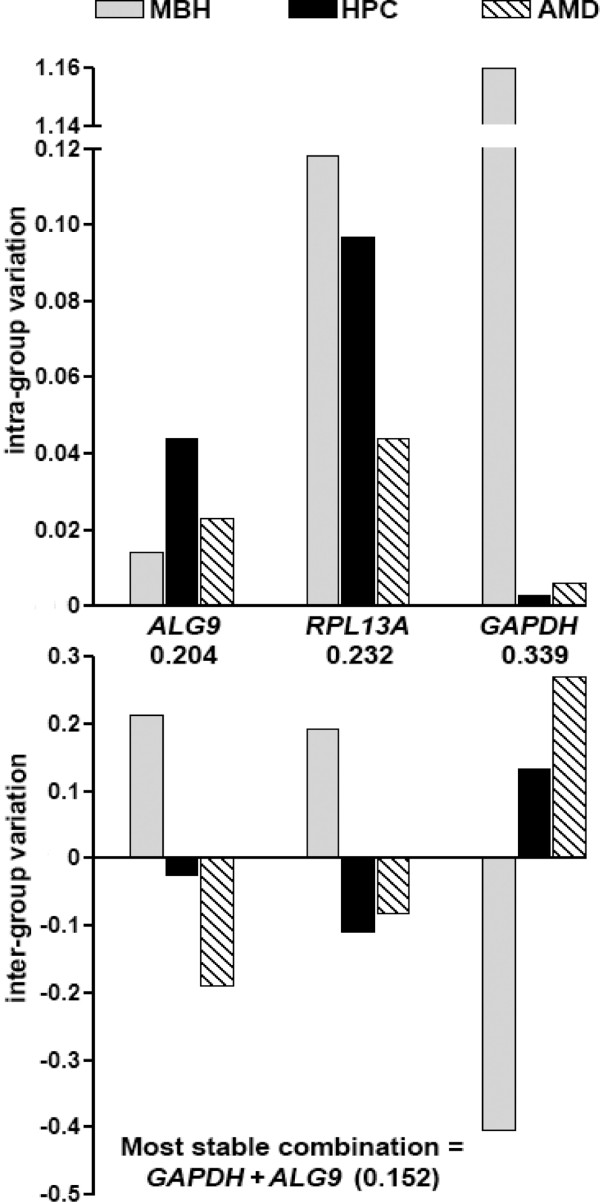
**NormFinder analysis for qRT-PCR prospective internal reference genes (qRT-PCR)**. Graphs show intra-group and inter-group variation [37] where gene expression quantity values were grouped according to brain region. Non-ordinate numbers show stability values for the given gene or gene combination.

In comparisons of inter-group and intra-group variation (genes grouped by brain region) *GAPDH *showed the highest variability in the MBH (Figures 3 and 4) with intra-group variation an order of magnitude higher than that observed for other genes.

### qRT-PCR. HRT NormFinder test of separate brain regions

NormFinder analysis of inter-group and intra-group variation highlighted the possibility that the most appropriate internal reference gene would vary according to brain region. For the MBH gene expression analysis, *ALG9 *was ranked as the most stably-expressed gene in all scenarios. i.e., when all nine genes were included, when only the five most stable genes were selected, or when selection was restricted to the three prospective internal reference genes (three most stably expressed genes). For the HPC and AMD gene expression analysis, *GAPDH *was ranked as the most stably-expressed gene, with the most stable combination being *GAPDH *and *RPL13A*. When only the three prospective internal reference genes were considered, *GAPDH *was again ranked as the most stably-expressed gene, with the best combination of *GAPDH *and *ALG9 *(Table 2).

### qRT-PCR. HRT NormFinder deviant sample test

Causes for observed differences in relative expression stability of the most stably expressed genes in the MBH compared to in the extra-hypothalamic regions (HPC and AMD) were elucidated by analysis of intra-group variation (Figures 3 and 4). As previously described, we tested the influence of this variation by omitting the sample contributing the most to MBH gene expression variability. With this omission, *GAPDH *showed the highest overall expression stability, and as before, stability rankings were identical with and without regional grouping considerations. In the omission test, *GAPDH *was also the most stable (0.219) when the 5 most stable genes were compared. The most stable combination was *GAPDH *+ *RPL13A *(0.137). However, when group comparisons were limited to prospective internal reference genes only, the most stable single gene was again *ALG9 *(0.171) with the most stable combination being *GAPDH *+ *ALG9 *(0.106).

### qRT-PCR. HRT BestKeeper analysis

In the analysis of all three brain regions, only the predicted housekeeping genes *GAPDH*, *RPL13A *and *ALG9 *met BestKeeper crossing point [38] standard deviation recommendations ((std dev [± CP]) < 1) for consideration as stably expressed genes. *RPL13A *displayed the lowest indices of variation (Table 3). Aside from the three prospective internal reference genes, *GAD1 *and *GABRA4 *showed the next highest expression stability. In light of findings from the NormFinder analysis, the MBH was analyzed separately from the extra-hypothalamic regions. For the separate MBH gene expression analysis, five genes met BestKeeper recommendations based on CP standard deviation for consideration as internal reference genes. They were ranked as follows, from lowest-to-highest variation (i.e., low variation indicates high stability): *GABRA1*, *ALG9*, *RPL13A*, *GABRA4 *and *GABRD*. However, when the most deviant MBH sample was omitted, *ALG9 *and *RPL13A *received highest (and equal) relative stability rankings followed by *GABRA1*, *GABRA4 *and *GABRE*. For the extra-hypothalamic HPC and AMD analysis, the five genes with lowest crossing point standard deviation from lowest to highest variation were *RPL13A*, *GAPDH*, *ALG9*, *GABRE *and *GABRD*.

**Table 3:** BestKeeper qRT-PCR descriptive statistics for internal reference genes

	*GAPDH*	*RPL13A*	*ALG9*
n	36	36	36
geometric mean [CP]	22.65	25.31	27.72
arithmetic mean [CP]	22.70	25.32	27.73
min [CP]	20.72	23.89	26.30
max [CP]	30.73	28.62	30.97
std dev [± CP]	0.88	0.49	0.63
CV [% CP]	3.86	1.95	2.27
min [x-fold]	-3.82	-2.42	-2.68
max [x-fold]	275.30	7.95	9.67
std dev [± x-fold]	1.84	1.41	1.55

n = number of samples; CP = Crossing point; std dev [± CP] = standard deviation of the CP; CV

[% CP] = variance coefficient expressed as a percentage of the CP level.

### Rhesus GeneChip^® ^HRT study. All probe sets grouped by region

Our search method (see methods) revealed 1444 probe sets on the rhesus macaque GeneChip^® ^annotated for the housekeeping genes [33]. When data was grouped by region, the most stably expressed probe set: MmugDNA.22897.1.S1_s_at (stability value 0.064) was annotated for *Homo sapiens *putative translation initiation factor (SUI1), mRNA. The most stable combination of probe sets was MmuSTS.4478.1.S1_at (multiple RefSeq IDs, annotated as "similar to guanine nucleotide binding protein, alpha stimulating activity polypeptide 1 isoform c") and MmugDNA.13670.1.S1_at (multiple RefSeq IDs) annotated for human aspartyl aminopeptidase (*DNPEP*). The range of stability values for all probe sets considered was 0.064-1.109, however expression levels for most of the most stably expressed probe sets were below levels of detection according to Microarray Suite version 5.0 (MAS 5.0) [39] analysis (Table 4).

**Table 4:** 20 most stably expressed probe sets across brain regions of macaques in HRT study

HRT: brain region					
rank/1444	stability value	descriptor "GEN = "	gene symbol	probe set ID	exp

1	0.064	*SUI1*	---	MmugDNA.22897.1.S1_s_at	+++
2	0.066	*VEGFB*	LOC722115	MmugDNA.8259.1.S1_at	-
3	0.073	1?	---	MmugDNA.15979.1.S1_at	-
4	0.073	*RPS14*	LOC697734	MmugDNA.3842.1.S1_at	-
5	0.074	*SDC3*	LOC704374	MmugDNA.32966.1.S1_at	-
6	0.074	*RPS27A*/ubiquitin	LOC697620/LOC701770	MmugDNA.43194.1.S1_at	-
7	0.074	2?	LOC693803	MmugDNA.23776.1.S1_at	-
8	0.076	*MAPKAPK2*	*MAPKAPK2*	MmugDNA.37463.1.S1_at	-
9	0.076	*RPS14*	LOC697734/LOC710901	MmugDNA.3842.1.S1_s_at	++++
10	0.076	*TADA3L*	*TADA3L*	MmugDNA.35773.1.S1_at	-
11	0.078	*ACVRL1*	*ACVRL1*	MmugDNA.35470.1.S1_at	-
12	0.078	*CALM1*	---	MmugDNA.30276.1.S1_s_at	-
13	0.079	*STK24*	---	MmugDNA.42176.1.S1_at	-
14	0.080	*GPAA1*	*GPAA1*	MmugDNA.10539.1.S1_s_at	+
15	0.080	*COL6A1*	---	MmugDNA.15324.1.S1_at	-
16	0.080	*EEF1A1*	LOC702809/LOC703715/LOC704199/LOC704438/LOC709017/LOC715351	MmuSTS.4277.1.S1_at	-
17	0.080	*HIST1H2BC*	LOC696506	MmunewRS.953.1.S1_x_at	-
18	0.080	*COX7C*	LOC693275/LOC702058/LOC706698	MmugDNA.43583.1.S1_x_at	+
19	0.081	*RAD9A*	LOC712345	MmugDNA.26624.1.S1_at	-
20	0.081	*API5*	*API5*	MmugDNA.34847.1.S1_at	-

**HRT: treatment**					

rank/1444	stability value	descriptor "GEN = "	gene symbol	probe set ID	exp

1	0.053	*ARHGEF7*	*ARHGEF7*	MmugDNA.4544.1.S1_at	-
2	0.054	*RPS27A*/ubiquitin	LOC697620/LOC701770	MmugDNA.43194.1.S1_at	-
3	0.056	*API5*	API5	MmugDNA.34847.1.S1_at	-
4	0.057	*HPCAL1*	---	MmugDNA.15147.1.S1_at	-
5	0.058	*GRIK5*	---	MmugDNA.24126.1.S1_at	-
6	0.059	*RPS25*	LOC719947	MmugDNA.41046.1.S1_at	-
7	0.062	*PFDN1*	LOC697795	MmugDNA.2620.1.S1_at	+++
8	0.062	*ATP5S*	LOC710290	MmugDNA.29002.1.S1_at	-
9	0.062	*EEF1A1*	LOC702809/LOC703715/LOC704199/LOC704438/LOC709017/LOC715351	MmuSTS.4277.1.S1_at	-
10	0.062	*FLJ10808*	LOC711751	MmugDNA.6835.1.S1_at	-
11	0.063	*GPAA1*	GPAA1	MmugDNA.10539.1.S1_s_at	+
12	0.063	HSP?	LOC708240/LOC709412	Mmu.13079.1.S1_at	-
13	0.064	*MCM3APAS*	---	MmugDNA.7848.1.S1_at	-
14	0.064	*RPL37*	---	MmugDNA.8209.1.S1_at	-
15	0.064	dJ612B15.1	LOC698967	MmunewRS.398.1.S1_at	++++
16	0.064	*SARS*	---	MmugDNA.37510.1.S1_at	-
17	0.064	*YWHAE*	LOC720295	MmugDNA.14868.1.S1_at	-
18	0.064	*RPS24*	LOC701477/LOC702961/LOC704054/LOC705596/LOC707085/LOC711145/LOC715668/LOC717801	MmugDNA.6998.1.S1_at	++++
19	0.064	*HIST1H2BC*	LOC696506	MmunewRS.953.1.S1_at	-
20	0.064	*ATP6V1F*	LOC705175	MmugDNA.5612.1.S1_at	-

1444 probe sets annotated for genes in the independently verified set were analyzed. Probe sets in the table are in order of most stably expressed to least stably expressed within the top 20. "descriptor" is the gene symbol most closely representing annotation at the time of table creation. Here "GEN = " annotations were prioritized. "---" = no gene symbol listed. "exp" = gene expression represented by hybridization signal intensity [52].

"+" and "-" symbols represent signal intensity values averaged across all animals and obtained using Affymetrix^® ^GCOS analysis, and global scaling to an average target intensity of 200. (-) = "undetectable", (+) = "signal intensity < 500", (++) = "signal intensity 500-1500", (+++) = "signal intensity 1501-10000". (++++) = "signal intensity > 10000".

824 "expressed sequences" from the original 1444 ('1c' in the methods) showed gene expression levels above the limits of detection (Table 5). When these were grouped by region the most stably expressed probe set: MmugDNA.22897.1.S1_s_at (stability value 0.065) was the same probe set that scored the highest expression stability rating when all 1444 sequences were considered (Tables 4 and 5). The most stably expressed combination of two probe sets (stability value 0.041) was MmugDNA.22897.1.S1_s_at and MmugDNA.33967.1.S1_at (not annotated). The range of stability values was 0.065-1.124.

**Table 5:** 20 most stably expressed probe sets detected in MAS 5.0 analysis of HRT study

HRT: brain region					
rank/824	stability value	descriptor "GEN = "	gene symbol	probe set ID	exp

1	0.065	*SUI1*	---	MmugDNA.22897.1.S1_s_at	+++
2	0.072	*B4GALT3*	LOC719920	MmugDNA.396.1.S1_at	+
3	0.086	*RPL13A*	LOC698713	MmugDNA.16774.1.S1_s_at	++++
4	0.086	*LDHB*	LDHB	MmugDNA.24833.1.S1_s_at	++++
5	0.088	*RPS14*	LOC697734/LOC710901	MmugDNA.3842.1.S1_s_at	++++
6	0.091	*RUVBL2*	---	MmugDNA.33967.1.S1_at	+
7	0.092	*EIF4A2*	*EIF4A2*	MmugDNA.25666.1.S1_at	+++
8	0.093	*RRAGA*	*BRI3*	MmugDNA.15747.1.S1_at	+++
9	0.096	*NDUFA1*	*NDUFA1*	MmugDNA.13559.1.S1_at	+++
10	0.099	*SNRPA*	*SNRPA*	MmugDNA.6187.1.S1_at	+
11	0.100	*PFDN1*	LOC697795	MmugDNA.2620.1.S1_at	+++
12	0.100	*PRPF8*	*PRPF8*	MmugDNA.21692.1.S1_at	++
13	0.102	*EMR2*	*EMR2*	MmuSTS.3377.1.S1_at	+
14	0.104	similar to calmodulin	LOC715270/LOC717686	MmunewRS.652.1.S1_at	++++
15	0.105	*CCND3*	CCND3	MmuSTS.1979.1.S1_at	+
16	0.106	*PSMD2*	LOC712473	MmuSTS.2326.1.S1_at	+++
17	0.106	similar to ACTG1	LOC713687	Mmu.5709.1.S1_x_at	++++
18	0.106	*UQCRQ*	*UQCRQ*	MmugDNA.18513.1.S1_s_at	+++
19	0.106	*NUDT3*	LOC718568	MmugDNA.22176.1.S1_at	++
20	0.107	*ARL2*	LOC722013	MmugDNA.11257.1.S1_s_at	+++

**HRT: treatment**					

rank/824	stability value	descriptor "GEN = "	gene symbol	probe set ID	exp

1	0.059	*SUI1*	---	MmugDNA.22897.1.S1_s_at	+++
2	0.063	*RUVBL2*	---	MmugDNA.33967.1.S1_at	+
3	0.068	*TMED9*	LOC702446	MmugDNA.32101.1.S1_s_at	++
4	0.071	*PSMD2*	LOC712473	MmuSTS.2326.1.S1_at	+++
5	0.072	*SARS*	LOC695046	MmugDNA.33979.1.S1_at	+++
6	0.073	*AHSA1*	*AHSA1*	MmugDNA.13760.1.S1_at	+++
7	0.074	*RAD23A*	LOC720675	MmugDNA.4491.1.S1_s_at	++
8	0.074	*TCFL1*	*VPS72*	MmuSTS.798.1.S1_at	+
9	0.074	*TALDO1*	LOC720828	MmugDNA.14258.1.S1_s_at	+++
10	0.075	*NDUFA7*	LOC711212	MmugDNA.17832.1.S1_at	++
11	0.077	*RRAGA*	*BRI3*	MmugDNA.15747.1.S1_at	+++
12	0.077	*RPS14*	LOC697734///LOC710901	MmugDNA.3842.1.S1_s_at	++++
13	0.077	*AKR1A1*	*AKR1A1*	MmugDNA.35875.1.S1_at	++
14	0.077	*CYB5R1*	LOC704519	MmuSTS.4187.1.S1_at	++
15	0.077	*COX7A2L*	LOC707203	MmugDNA.24749.1.S1_at	+
16	0.077	*COX7A2L*	LOC707203	MmugDNA.24749.1.S1_at	+
17	0.078	*UBB*	LOC697557	MmugDNA.35404.1.S1_at	++++
18	0.078	*NDUFS5*	*NDUFS5*	MmugDNA.29879.1.S1_at	+++
19	0.079	*COX4I1*	LOC714951	MmugDNA.39322.1.S1_s_at	+++
20	0.080	*PFDN1*	LOC697795	MmugDNA.2620.1.S1_at	+++

824 probe sets were selected from the 1444 HRT probe sets annotated for housekeeping genes based on MAS 5.0 signal intensity and detection call metrics. Probe sets in the table are in order of most stably expressed to least stably expressed within the top 20. "descriptor" is the gene symbol most closely representing annotation at the time of table creation. Here "GEN = " annotations were prioritized. "---" = no gene symbol listed. "exp" = gene expression represented by hybridization signal intensity [52].

"+" and "-" symbols represent signal intensity values averaged across all animals and obtained using Affymetrix^® ^GCOS analysis, and global scaling to an average target intensity of 200. (-) = "undetectable", (+) = "signal intensity < 500", (++) = "signal intensity 500-1500", (+++) = "signal intensity 1501-10000". (++++) = "signal intensity > 10000".

### Rhesus GeneChip^® ^HRT study. All probe sets grouped by treatment

Using the 1444 probe sets annotated for housekeeping genes, when data were grouped by treatment, the most stably expressed (stability value = 0.053) probe set (MmugDNA.4544.1.S1_at, RefSeq ID XR_010342) was annotated for Rho guanine nucleotide exchange factor 7 (*ARHGEF7*). The most stably expressed combination of two probe sets (0.038) was for MmugDNA.30089.1.S1_x_at [RefSeq: XR_010595]; similar to Somatostatin receptor type 5) and MmunewRS.652.1.S1_at (multiple RefSeq transcript IDs; similar to calmodulin 1). The range of stability values for all probe sets considered was 0.053-0.396, however, expression levels for many probe sets were below levels of detection using MAS 5.0 analysis (Table 4).

When only the 824 "expressed sequences" from the original 1444 were grouped by treatment ('1c' in the methods, Table 5), the most stably expressed sequence was the same probe set, MmugDNA.22897.1.S1_s_at (stability value 0.059), that was most stably expressed when analysis grouping was done according to region. The most stably expressed combination of two probe sets (stability value 0.038) was the aforementioned MmugDNA.22897.1.S1_s_at and MmugDNA.18192.1.S1_at [RefSeq: XM_001099724], similar to NADH dehydrogenase (ubiquinone) flavoprotein 2, 24 kDa). The range of stability values was 0.059-0.395.

### Human GeneChip^® ^cycle study. All probe sets grouped by brain region

Our search method (see methods) revealed 1433 probe sets annotated for housekeeping genes on the human GeneChip^®^. When all probe sets were compared by brain region, the most stably expressed (stability value = 0.041) was probe set 213214_x_at [RefSeq: NM_001614], which represented actin, gamma 1 (*ACTG1*). The most stably expressed combination of probe sets, was 216295_s_at with multiple RefSeq transcript IDs for clathrin, light chain (*CLTA*) together with 202021_x_at, [RefSeq: NM_005801] representing eukaryotic translation initiation factor 1 (*EIF1*). The range of stability values was 0.041-0.987. Four probe sets annotated for *ACTG1 *were among the top 20 most stably expressed of the 1433 evaluated, and all four of these showed high expression (Table 6).

**Table 6:** most stably expressed probe sets across brain regions of normally menstruating macaques

Cycle study: brain region				
rank/1433	stability value	gene symbol	probe set ID	exp

1	0.041	*ACTG1*	213214_x_at	+++
2	0.048	*ACTG1*	221607_x_at	++++
10	0.062	*ACTG1*	224585_x_at	++++
17	0.066	*ACTG1*	201550_x_at	+++
4	0.054	*CALM1/CALM2/CALM3*	207243_s_at	++++
19	0.067	*CLTA*	216295_s_at	+++
8	0.060	*COX7C*	201134_x_at	++
9	0.060	*DIAPH1*	1560080_at	-
16	0.065	*EIF1*	212227_x_at	+++
5	0.056	*GPX4*	201106_at	+++
15	0.063	*H6PD*	221892_at	+
20	0.067	*LDHB*	213564_x_at	+++
13	0.062	*MVK*	36907_at	++
12	0.062	*NACAP1*	211445_x_at	+++
6	0.059	*POLR2A*	217420_s_at	-
7	0.059	*RAC1*	1567457_at	-
11	0.062	*SAFB*	213635_s_at	-
18	0.067	*TCF25*	221495_s_at	-
3	0.052	*YWHAH*	236559_at	-
14	0.063	*ZNF592*	227507_at	-
				

**Cycle study: cycle phase**				

rank/1433	stability value	gene symbol	probe set ID	exp

2	0.025	*ACTG1*	221607_x_at	++++
4	0.026	*ACTG1*	213214_x_at	+++
9	0.029	*ACTG1*	224585_x_at	++++
15	0.030	*ACTG1*	212988_x_at	+++
16	0.030	*ACTG1*	201550_x_at	+++
11	0.029	*ARHGEF7*	236416_at	-
1	0.024	*CLTA*	216295_s_at	+++
8	0.028	*CLTA*	200960_x_at	+++
17	0.031	*COPS6*	201405_s_at	+++
10	0.029	*COX7C*	201134_x_at	++
6	0.028	*CSNK2B/LY6G5B*	201390_s_at	+++
18	0.031	*EEF2*	200094_s_at	+++
3	0.026	*EIF1*	202021_x_at	+++
7	0.028	*EIF1*	212130_x_at	+++
12	0.029	*EIF1*	212227_x_at	+++
19	0.031	*GPAA1*	215690_x_at	++
13	0.030	*HINT1*	207721_x_at	+++
14	0.030	*PPP2CB*	201375_s_at	+++
5	0.027	*RPS27A/UBB/UBC*	200633_at	++++
20	0.031	*SNX3*	208781_x_at	+++

1433 probe sets annotated for genes in the independently verified set were analyzed. Probe sets in the table are presented here in alphabetical groupings by annotation irrespective of rank within the top 20. "exp" = gene expression represented by hybridization signal intensity [52].

"+" and "-" symbols represent signal intensity values averaged across all animals and obtained using Affymetrix^® ^GCOS analysis, and global scaling to an average target intensity of 200. (-) = "undetectable", (+) = "signal intensity < 500", (++) = "signal intensity 500-1500", (+++) = "signal intensity 1501-10000". (++++) = "signal intensity > 10000".

### Human GeneChip^® ^cycle study. All probe sets grouped by cycle phase

Using the 1433 probe sets annotated for housekeeping genes, when all probe sets were grouped for comparison according to menstrual cycle phase, the most stably expressed (stability value = 0.024) probe set was 216295_s_at, which had multiple RefSeq transcript ID annotations for clathrin, light chain (*CLTA*). The most stable combination of probe sets (stability value = 0.017) was the aforementioned 216295_s_at combined with 221607_x_at [RefSeq: NM_001614], which represented actin, gamma 1 (*ACTG1*). The range of stability values for the 1433 evaluated probe sets was 0.024-0.373. Five probe sets annotated for *ACTG1 *were among the top 20 most stably expressed of the 1433 evaluated, and all four of these showed high expression (Table 6).

### Human GeneChip^® ^cycle study. Most representative probe sets grouped by brain region

Using criteria described in the methods for representative probe set selection, our search method revealed 544 probe sets. When comparisons were made across brain regions, the most stably expressed sequence (stability value 0.053) was a probe set (207243_s_at, NCBI representative public ID NM_001743) representing *Homo sapiens *calmodulin 2 (phosphorylase kinase, delta) (*CALM2*), mRNA and annotated as calmodulin 1-3 (*CALM1 */*CALM2 */*CALM3*) on NetAffx. Sequences representing the most stably expressed combination of two genes (stability value = 0.030) were probe sets 201405_s_at [RefSeq: NM_006833] and 200633_at [RefSeq: NM_018955] which represent *COP9 *constitutive photomorphogenic homolog subunit 6 (*COPS6*) and ubiquitin B (*UBB*) respectively The latter probe set, 200633_at, was also annotated for ribosomal protein S27a (*RPS27A*) and ubiquitin C (*UBC*) (Table 7).

**Table 7:** 20 most stably expressed of the "most representative" probe sets for housekeeping genes across brain regions of normally menstruating macaques

Cycle study: brain region				
rank/544	stability value	gene symbol	probe set ID	exp

1	0.053	*CALM1/CALM2/CALM3*	207243_s_at	++++
2	0.060	*GPX4*	201106_at	+++
3	0.064	*COX7C*	201134_x_at	++
4	0.068	*COPS6*	201405_s_at	+++
5	0.069	*NDUFS5/RPL10*	201757_at	++
6	0.071	*VPS72*	202261_at	++
7	0.071	*RPS27A/UBB/UBC*	200633_at	++++
8	0.073	*ATP6V1F*	201527_at	+++
9	0.074	*SSR2*	200652_at	++
10	0.075	*PPP2CB*	201375_s_at	+++
11	0.076	*HINT1*	207721_x_at	+++
12	0.076	*CSNK2B/LY6G5B*	201390_s_at	+++
13	0.078	*PFDN5*	207132_x_at	+++
14	0.078	*SPAG7*	200053_at	++
15	0.078	*RPL10*	200725_x_at	+++
16	0.078	*GP2*	206681_x_at	-
17	0.080	*RPLP1*	200763_s_at	+++
18	0.081	*UQCRH*	202233_s_at	+++
19	0.081	*COX7A2L*	201256_at	+++
20	0.083	*ATF4*	200779_at	++

**Cycle study: cycle phase**				

rank/544	stability value	gene symbol	probe set ID	exp

1	0.026	*CSNK2B/LY6G5B*	201390_s_at	+++
2	0.027	*RPS27A/UBB/UBC*	200633_at	++++
3	0.027	*HINT1*	207721_x_at	+++
4	0.027	*COPS6*	201405_s_at	+++
5	0.028	*PPP2CB*	201375_s_at	+++
6	0.028	*COX7C*	201134_x_at	++
7	0.029	*ATP6V1F*	201527_at	+++
8	0.030	*HAX1*	201145_at	++
9	0.031	*GPX4*	201106_at	+++
10	0.031	*CALM1/CALM2/CALM3*	207243_s_at	++++
11	0.031	*SPAG7*	200053_at	++
12	0.031	*GPAA1*	201618_x_at	+
13	0.032	*PSMD2*	200830_at	+++
14	0.032	*COX7A2L*	201256_at	+++
15	0.033	*CLTA*	204050_s_at	+++
16	0.033	*NDUFA2*	209224_s_at	+++
17	0.033	*SSR2*	200652_at	++
18	0.034	*TTC1*	201434_at	++
19	0.034	*RPLP1*	200763_s_at	+++
20	0.034	*INPP5K*	202782_s_at	-

Probe sets are listed according to rank. Higher rank indicates greater expression stability. Note that selection was narrowed to 544 probe sets likely to be most representative of genes of interest.

"exp" = gene expression represented by hybridization signal intensity [52].

"+" and "-" symbols represent signal intensity values averaged across all animals and obtained using Affymetrix^® ^GCOS analysis, and global scaling to an average target intensity of 200. (-) = "undetectable", (+) = "signal intensity < 500", (++) = "signal intensity 500-1500", (+++) = "signal intensity 1501-10000". (++++) = "signal intensity > 10000".

### Human GeneChip^® ^cycle study. Most representative probe sets grouped by cycle phase

Using the 544 "most representative" probe sets, when comparisons were made across phases of the menstrual cycle, the most stably expressed sequence (stability value = 0.026) was a probe set, 201390_s_at [RefSeq: NM_001320], representing casein kinase 2, beta polypeptide (*CSNK2B*) and annotated for lymphocyte antigen 6 complex, locus G5B (*LY6G5B*). The most stably expressed combination of two genes (stability value = 0.019) was represented by the aforementioned *CSNK2B *probe set (201390_s_at) and a probe set, 200633_at [RefSeq:NM_018955] representing ubiquitin B (*UBB*) and annotated for ribosomal protein S27a (*RPS27A*), and ubiquitin C (*UBC*) (Table 7). Thirteen probe sets among the 20 most stably ranked across cycle phase were also among the 20 most stably ranked across brain region.

### Human GeneChip^® ^cycle study. Most representative probe sets: comparison between popular normalizers

We refer to the "popular normalizers" as the thirteen widely used normalizers http://www.compugen.co.il/supp_info/Housekeeping_genes.html in addition to Asparagine-linked glycosylation protein 9 homolog (*ALG9*) and Ribosomal protein L13a (*RPL13A*). When the most representative probe sets for the popular normalizers were compared, the probe set 200017_at [RefSeq:NM_002954], representing ribosomal protein S27a (*RPS27A*) was the most stably expressed (Table 8). This finding was consistent when comparisons were made across brain region or phase of the menstrual cycle. This probe set was also annotated for ubiquitin B (*UBB*) and ubiquitin C (*UBC*). In the analysis grouped by brain region, seven probe sets in the top 20 showed expression below limits of detection, whereas with the phase grouping, expression levels for one probe set out of 20 were below limits of detection (Table 8).

**Table 8:** Most stably expressed probe sets annotated for popular normalizing genes

**Cycle study: brain region**					**HRT: brain region**				
	
rank/544	stability value	annotation target	probe set ID	exp	rank/1444	stability value	annotation target	probe set ID	exp
	
76	0.109	*RPS27A*	200017_at	++++	6	0.074	*RPS27A*	MmugDNA.43194.1.S1_at	-
182	0.141	*RPL11*	200010_at	+++	23	0.082	*RPL13A*	MmugDNA.16774.1.S1_s_at	++++
216	0.151	*ALG9*	228817_at	+	62	0.091	*RPL32*	MmugDNA.25831.1.S1_s_at	++++
217	0.152	*RPL19*	200029_at	+++	187	0.112	*LDHA*	MmugDNA.40348.1.S1_at	-
233	0.159	*ARHGDIA*	201168_x_at	++	350	0.135	*ACTB*	AFFX-Mmu-actin-3_s_at	++++
236	0.160	*ACTB*	200801_x_at	++++	382	0.140	*GAPDH*	AFFX-Mmu-gapdh-3_x_at	++++
261	0.169	*RPS18*	201049_s_at	++++	383	0.140	*PGK1*	MmugDNA.17381.1.S1_at	-
281	0.178	*RPL13A*	200716_x_at	++++	535	0.161	*RPL19*	MmugDNA.25770.1.S1_at	+++
333	0.201	*GAPDH*	212581_x_at	++++	628	0.182	*ARHGDIA*	MmugDNA.2575.1.S1_at	-
352	0.211	*HSPCB*	200064_at	++++	825	0.231	*HSPCB*	MmugDNA.32274.1.S1_at	+++
363	0.220	*RPL32*	200674_s_at	++++	842	0.235	*RPS18*	MmugDNA.43260.1.S1_at	++++
389	0.240	*PGK1*	200737_at	++	934	0.262	*ALG9*	MmugDNA.30425.1.S1_at	+
454	0.293	*NONO*	200057_s_at	+++	1129	0.329	*NONO*	MmugDNA.18264.1.S1_at	++
456	0.294	*ALDOA*	200966_x_at	+++	1271	0.410	*RPL11*	MmugDNA.25225.1.S1_at	+
464	0.302	*LDHA*	200650_s_at	++					
									
	
**Cycle study: cycle phase**					**HRT: treatment**				
	
rank/544	stability value	annotation target	probe set ID	exp	rank/1444	stability value	annotation target	probe set ID	exp
	
41	0.039	*RPS27A*	200017_at	++++	2	0.054	*RPS27A*	MmugDNA.43194.1.S1_at	-
87	0.045	*RPL11*	200010_at	+++	25	0.066	*RPL32*	MmugDNA.25831.1.S1_s_at	++++
110	0.048	*RPL19*	200029_at	+++	143	0.078	*GAPDH*	AFFX-Mmu-gapdh-3_x_at	++++
123	0.049	*ACTB*	200801_x_at	++++	164	0.079	*RPL13A*	MmugDNA.16774.1.S1_s_at	++++
150	0.051	*ARHGDIA*	201168_x_at	++	215	0.082	*ACTB*	AFFX-Mmu-actin-3_s_at	++++
182	0.053	*RPL13A*	200716_x_at	++++	294	0.086	*PGK1*	MmugDNA.17381.1.S1_at	-
204	0.056	*RPS18*	201049_s_at	++++	350	0.089	*RPL19*	MmugDNA.25770.1.S1_at	+++
253	0.060	*ALG9*	228817_at	+	463	0.095	*ARHGDIA*	MmugDNA.2575.1.S1_at	-
338	0.068	*GAPDH*	212581_x_at	++++	568	0.099	*RPS18*	MmugDNA.43260.1.S1_at	++++
347	0.069	*HSPCB*	200064_at	++++	571	0.099	*HSPCB*	MmugDNA.34686.1.S1_at	+++
361	0.073	*RPL32*	200674_s_at	++++	607	0.102	*LDHA*	MmugDNA.8511.1.S1_at	-
436	0.090	*ALDOA*	200966_x_at	+++	668	0.105	*ALG9*	MmugDNA.30425.1.S1_at	+
437	0.090	*NONO*	200057_s_at	+++	1212	0.168	*RPL11*	MmugDNA.25225.1.S1_at	+
478	0.104	*LDHA*	200650_s_at	++	1346	0.218	*NONO*	MmugDNA.770.1.S1_at	+
492	0.112	*PGK1*	200737_at	++					

Probe sets are listed according to rank. Higher rank indicates greater expression stability. Denominator in the rank column indicates the number of probe sets analyzed using criteria described in methods. Note that selection was narrowed to the 544 probe sets likely to be most representative of genes of interest where menstruating macaques were examined using the human GeneChip^®^. All 1444 probe sets that were annotated for genes of interest on the rhesus GeneChip^® ^are represented in the table.

"exp" = gene expression represented by hybridization signal intensity [52].

"+" and "-" symbols represent signal intensity values averaged across all animals and obtained using Affymetrix^® ^GCOS analysis, and global scaling to an average target intensity of 200. (-) = "undetectable", (+) = "signal intensity < 500", (++) = "signal intensity 500-1500", (+++) = "signal intensity 1501-10000". (++++) = "signal intensity > 10000"

### Findings common to both (HRT and cycle) studies

**Figure 5:**
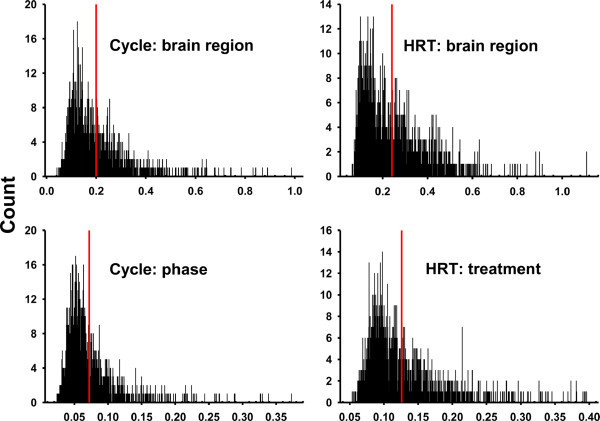
**Frequency distributions of relative expression stability values**. Histograms show counts on the ordinate axis and expression stability values on the abscissa. Histograms are arranged according to study (first word) and analysis grouping scheme (second word). 1433 and 1444 probe set were used in the cycle and HRT studies respectively to represent genes likely to be reliably expressed under multiple conditions (housekeeping genes). Red lines show average expression stability values.

In both studies, frequency distributions of expression stability data for the housekeeping gene probe sets were skewed toward the more stably expressed sequences (Figure 5), failing Kolmogorov-Smirnov (P < 0.001) and Shapiro-Wilks (P < 0.001) tests for normality. For the popular normalizers in both studies, probe sets for *RPS27A *tended to clump according to expression stability among the most stably expressed in the collective set (Table 8).

In six of the analyses conducted between the two experiments, 31 gene annotations were found to be common to multiple analyses, and 12 annotations were common to both experiments (Table 9). Probe sets annotated for *RPS27A*/ubiquitin were the most commonly observed stably expressed sequences in all analyses between the two studies.

**Table 9:** Gene annotations common across multiple experiments and analysis groupings

	HRT study: 20 most stably expressed				Cycle study: 20 most stably expressed			
		
gene annotation	1b: all probe sets (1444)		1c: "expressed" probe sets (824)		2a: all probe sets (1433)		2b: representative probe sets (544)	
		
	brain region	hormone trt	brain region	hormone trt	brain region	cycle phase	brain region	cycle phase
*ACTG1*			√^†^		√	√		
*API5*	√	√						
*ARHGEF7*		√				√		
*ATP6V1F*		√					√	√
*BRI3/RRAGA*			√	√				
*CALM1/CALM2/CALM3*	√*		√^†^		√		√	√
*CLTA*					√	√		√
*COPS6*						√	√	√
*COX7A2L*				√			√	√
*COX7C*	√				√	√	√	√
*CSNK2B/LY6G5B*						√	√	√
*EEF1A1*	√	√						
*EIF1*					√	√		
*GPAA1*	√	√				√		√
*GPX4*					√		√	√
*HINT1*						√	√	√
*HIST1H2BC*	√	√						
*LDHB*			√		√			
*NDUFS5*				√			√	
*PFDN1*		√	√	√				
*PPP2CB*						√	√	√
*PSMD2*			√	√				√
*RPLP1*							√	√
*RPS14*	√		√	√				
*RPS27A*/ubiquitin	√	√		√^#^		√	√	√
*RUVBL2*			√	√				
*SARS*		√		√				
*SPAG7*							√	√
*SSR2*							√	√
*SUI1*	√		√	√				
*VPS72*				√			√	

Numbers in parentheses show the total number of probe sets in the group under consideration. Check marks (√) show studies (HRT vs. cycle), probe set selection methods ("all ", "expressed", or "most representative" probe sets annotated for housekeeping genes) and analysis groupings (brain region, hormone treatment or cycle phase) under which the listed gene annotation appears.

* = *CALM1 *only. # = *UBB *only. † = "Similar to".

## Discussion

### qRT-PCR

Using three popular algorithms, we assessed relative gene expression stability across the three brain regions, to identify genes that met or exceeded criteria commonly used for selection of internal reference genes used for qRT-PCR normalization [8,16]. In each case the prospective internal reference genes *RPL13A*, *GAPDH *and *ALG9 *were found to be more reliable normalizers than the selected genes for GABAergic system components [26], with the exception was *GAD1*, which was ranked as being more stably expressed than *GAPDH *using geNorm. We suspected that this stems from the variation in *GAPDH *expression in the MBH, which the NormFinder intra-group analysis showed to be an order of magnitude higher than the variation in the expression of other examined genes. Reduction of sample variance, by removal of the most deviant MBH value produced a geNorm ranking where *GAPDH *was more highly ranked than *GAD1*.

The finding that higher gene expression variability occurred in the MBH compared to the extra-hypothalamic regions (HPC and AMD) was consistent with findings from a principal components (statistical) analysis of gene expression during phases of the rhesus macaque menstrual cycle [40] under conditions of the current study.

### Microarray evaluation

Probe sets annotated for *RPS27A*/ubiquitin were stably expressed in both studies and provide basis for comparing results in the human and rhesus GeneChips^®^. Note that in analysis of cycle study data grouped by region, probe sets for *RPS27A*/ubiquitin do not appear in the top 20 most stably expressed probe sets when all probe sets were considered (Table 6). However the *RPS27A*/ubiquitin probe set '200633_at' appears as the 26^th ^most stably expressed of the 1433 probe sets tested, and was extremely highly expressed with a rating of "++++" (data not shown). Comparatively, in the HRT study using the rhesus GeneChip^®^, the probe set 'MmugDNA.43194.1.S1_at' was annotated as "similar to ubiquitin and ribosomal protein S27A", and showed expression levels below MAS 5.0 analysis detection limits (Table 8). However another similarly annotated probe set (MmugDNA.26506.1.S1_at) was highly expressed, had a regional expression stability of 0.15 (rank = 453/1444) and a treatment expression stability of 0.069 (rank = 46/1444) (Data not shown). The rhesus macaque annotations for both of these probe sets are based on a *Macaca fascicularis *sequence annotated by comparison to the human sequence listed under RefSeq ID [NM_002954.3].

A detailed examination of the NetAffx annotation did not clarify if these probe sets interrogate the *RPS27A *sequence solely, or the *RPS27A*/ubiquitin fusion sequence. Note that ubiquitin is covalently bound to proteins targeted for posttranslational modification or degradation, and influences the intracellular localization and stability of proteins. The ubiquitin gene can be fused to a ribosomal protein gene, and this fusion gene may be referenced by *RPS27A *probe sets in the on the Affymetrix^® ^arrays.

Affymetrix^® ^arrays contain redundant probe sets that interrogate different regions of the same gene [41], and reflect differential regulation of alternative script production based on alternative splicing or polyadenylation [42]. Screening using qualitative present vs. absent calls from MAS5.0 analysis can improve reliability of evaluation among redundant probe sets [41,43,44].

Probe sets for the same gene commonly show differences in expression levels [41,43-45]. For well annotated genes like *ACTB*, these probe set sequences are likely to be accurate, and microarray analysis may be detecting variations in expression stability along different portions of the same sequence [44,46]. Although annotation of rhesus GeneChip^® ^probe sets are based on sequences defined using the human GeneChip^® ^as a template [47], annotation of many probe sets for the rhesus GeneChip^® ^are inferred and are frequently separated from verified sequences by more levels of interpretation than is the case for the human GeneChip^® ^[48]. For the rhesus macaque, annotation reliability of the subset of the rhesus GeneChip^® ^probe sets that have been verified using rhesus macaque tissues may exceed the reliability of probe sets annotated on the human GeneChip^®^. However full annotation of the rhesus GeneChip^® ^is still a work in progress [44] and to date, large numbers of functional rhesus macaque transcripts may be more reliably interrogated using the human GeneChip^®^.

### Robust normalizers identified by microarray analysis

In the cycle study, the occurrence of four highly expressed, well annotated *ACTG1 *probe sets ranked among the top 20 most stably expressed sequences in both analytical groupings, causes *ACTG1 *to meet criteria for what we propose to call a "robust normalizer". i.e., multiple segments of the same gene [44,46] show high expression stability across all experimental conditions.

Identification of a robust normalizer (*ACTG1*) was accomplished using the human but not the rhesus GeneChip^®^. In contrast, probe sets annotated for *RPS27A *showed high stability across all tissues and conditions used in both experiments (Tables 7, 8 and 9). However, annotation on the rhesus GeneChip^® ^was insufficient for exploration of robustness as a potential normalizer.

Twelve annotations from among the various selections of "20 most stably expressed" were common to both experiments (Table 9). Within the cycle study, multiple probe sets for the clathrin light chain (*CLTA*) were observed among the 20 most stably expressed under two methods of probe set selection, and were robustly expressed using MAS 5.0 detection (Tables 6 and 9).

### Popular normalizers

In both studies, the expression stabilities of popular normalizers were spread across much of the range observed in the overall pool of housekeeping genes. As a group, the popular normalizers did not show tendencies to be more stably expressed than the larger pool of housekeeping genes (See ranks in Table 8).

**Figure 6:**
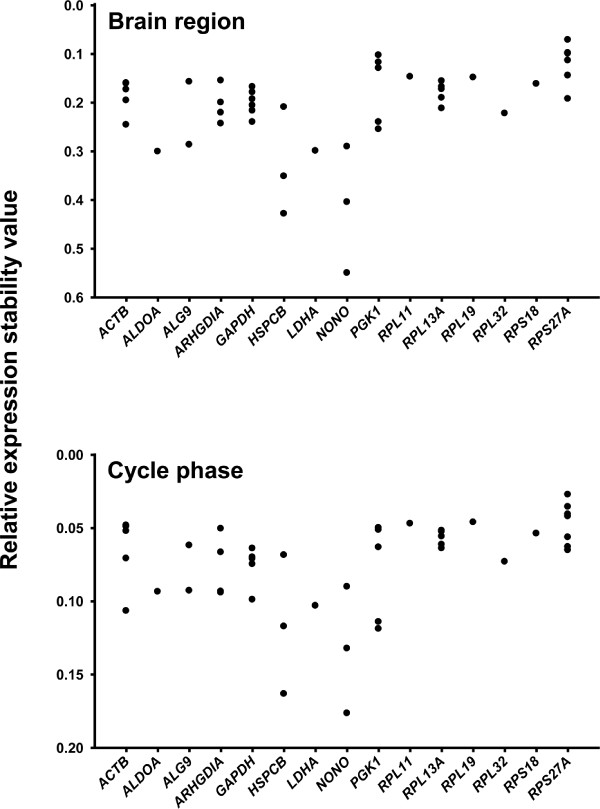
**Relative expression stability of 'popular normalizers' in the cycle study**. Relative expression stability of genes commonly used as internal references for normalization (popular normalizers). Each dot represents one probe set. Data from normally menstruating rhesus macaques. Values closer to zero (top of histogram) indicate greater expression stability. Microarray data from the amygdala, hippocampus and arcuate hypothalamus of normally menstruating rhesus macaques evaluated using the Affymetrix^® ^human GeneChip^®^. The chart shows expression stabilities of 45 probe sets representing 15 popular genes. The ordinate axis shows expression stability rank relative to expression stabilities of 1433 probe sets representing 575 human genes reliably expressed under multiple conditions. Gene expression stability values were generated from gene expression values grouped according to brain region or menstrual cycle phase using NormFinder software.

Probe sets for *RPS27A *and *RPL11 *showed high stability rankings among the popular normalizers for all analyses in both studies (Table 8). In analysis of the most representative probe sets for the cycle study, the appearance of clusters (by rank) of probe sets for popular normalizers *RPS27A*, *RPL13A*, *ARHGDIA*, *ACTB *and *GAPDH *showing high expression stability (Figure 6) under both regional and phase-based analyses prompted us to identify these genes as robust normalizers among the subset of popular normalizers.

**Figure 7:**
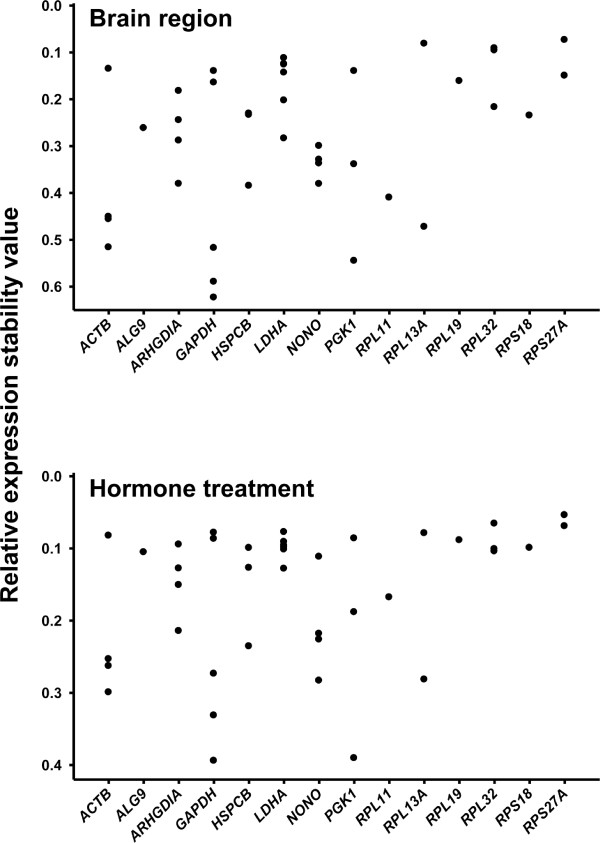
**Relative expression stability of 'popular normalizers' in the HRT study**. Relative expression stability of genes commonly used as internal references for normalization (popular normalizers). Each dot represents one probe set. Data from ovariectomized rhesus macaques receiving hormone replacement. Values closer to zero (top of histogram) indicate greater expression stability. Microarray data from the amygdala, hippocampus and arcuate hypothalamus of ovariectomized rhesus macaques receiving E_2 _or E_2_+ P_4 _and evaluated using the Affymetrix^® ^rhesus GeneChip^®^. The chart shows expression stabilities of 39 probe sets representing 15 popular genes. The ordinate axis shows expression stability rank relative to expression stabilities of 1444 probe sets representing 575 human genes reliably expressed under multiple conditions. Gene expression stability values were generated from gene expression values grouped according to either brain region or treatment group using NormFinder software.

Probe sets for *ACTB *and *GAPHD *are well annotated and used as controls on the rhesus GeneChip^® ^[44], however probe sets for these genes show a high range of expression stabilities (Figure 7). The significance of variable expression among well annotated probe sets for the same gene is under evaluation [41,44]. Possibly related to this probe set variability in expression and expression stability, we found sequences for *ACTB *and *GAPDH *to be problematic in qRT-PCR normalization of the HRT study (unpublished data).

### Comparison between qRT-PCR and microarray data

Under all selection criteria used in the current study, we indentified probe sets for genes more stably expressed than probe sets for genes (*RPL13A*, *ALG9 *and *GAPDH*) that we showed to be well suited for qRT-PCR normalization based on high concordance with microarray results, as well as relative expression stability comparisons using three algorithms designed for this purpose [26]. Interestingly, when all rhesus GeneChip^® ^probe sets were restricted to those for popular normalizers, *RPL13A *and *GAPDH *were among the more highly ranked (stably expressed) and showed high expression (Table 8). Additional genes may be open for consideration given that many probe sets on the rhesus GeneChip^® ^were more stably expressed than the probe sets for *RPL13A*, *ALG9 *and *GAPDH*. In addition, the probes used for qRT-PCR were derived from the same NCBI sequences as the rhesus GeneChip^® ^probe sets. The noticeable rank separation of *ALG9 *from *RPL13A *and *GAPDH *in the HRT study emphasizes the importance of considering that all genes included in the NormFinder analysis influence systemic variation which affects relative ranking [37].

Note that how representative we considered a probe set to be was based on the cumulative record of annotation history associated with the probe set. Other investigators performing gene expression comparisons using approximately 500 genes showed high correlation between results from multiple microarray platforms (Affymetrix, Agilent, Illumina, GE Healthcare and NCI) and TaqMan^® ^qRT-PCR results. In addition, high correlations between qRT-PCR and microarray results using RMA-normalized log 2-transformed data in nonhuman primates have been demonstrated elsewhere [49]. The use of publicly available microarray data provides a selection method that is independent of the microarray platform or normalization methodology, and is able to cope with gene lists that overlap only partially [22]. We considered genes showing both low and high expression in addition to high expression stability because the data range between the minimum and maximum expression levels could have a profound influence on normalizer calibration. Although pairwise gene expression stability measures account for effects of large differences in expression levels [34], having a small range of control gene expression values can skew comparative detection sensitivity.

It is important to note that other methods would likely detect stably expressed genes from the current study which are not included in the published set of 575 human housekeepers. However, our goal was for current study to identify candidates likely enough to be stably expressed over experimental variation so as to allow us to best manage PCR for future macaque studies involving these brain regions, where additional microarray analysis may not be feasible.

## Conclusions

Using gene microarray analysis of two experiments, we ranked expression stability of sequences for 575 published housekeeping genes under six sex-steroid environments in three regions of the rhesus macaque brain. Sequences for popularly used normalizing genes did not tend to be more stably expressed than sequences from the overall pool of housekeeping genes, and probe set expression stability values for multiple genes frequently overlapped. Although annotation quality of the rhesus GeneChip^® ^was not sufficient to facilitate detection of robust normalizers, comparisons of relative expression stability values for the same genes between the rhesus and human GeneChips^® ^were possible. Robust normalizer candidates were identified using the human GeneChip^®^. For example, in the cycle study, multiple probe sets for *ACTG1 *were stably expressed under all conditions in all brain regions at levels allowing easy detection. Therefore we recommend *ACTG1 *as a robust normalizer for examination of MBH, HPC and AMD under changing ovarian hormonal conditions. Comparatively, in both studies, probe sets for *RPS27A *were stably expressed under all conditions in all three brain regions at levels allowing easy detection, and may be the normalizer of choice for MBH, HPC and AMD comparison between both experiments. However, further examination of probe set annotation quality for this gene is recommended.

In summary, we identified multiple genes that were more stably expressed across more experimental conditions and showed less probe set expression variability than popularly used genes meeting typical requirements for qRT-PCR normalization. These findings demonstrate uses for well annotated gene microarray data in addressing widely documented problems associated with the selection of experimentally specific internal reference genes.

## Methods

### Overview (Figure 1)

Tissue from the arcuate nucleus of the medial basal hypothalamus (MBH), hippocampus (HPC) and amygdala (AMD) of rhesus macaques *Macaca mulatta *were collected for examination in two separates studies. One study was designed to assess effects of three hormone replacement therapy (HRT) conditions on gene expression in the three brain regions collected, and this gene expression was examined using the rhesus GeneChip^®^. In the HRT study qRT-PCR was conducted on all tissues in addition to microarray analysis. In a second study (cycle study), gene expression in the three brain regions during early follicular (EF) late follicular (LF) and mid luteal (ML) phases of the menstrual cycles of normally menstruating macaques was examined using the human GeneChip^®^.

In the gene microarray analyses for both the HRT and cycle studies, comparisons of the relative expression stabilities of probe sets annotated for independently assessed human housekeeping genes [33] were conducted using NormFinder [31] algorithms equipped to process data from microarrays. Because the reliability of the NormFinder algorithm is dependent on the expression stability of the sequences examined [37], the most widely used normalizers from the list of housekeeping genes http://www.compugen.co.il/supp_info/Housekeeping_genes.html, as well as *RPL13A *and *ALG9 *(the latter two considered noteworthy based on our prior results [26] and considered here as part of the "popular normalizer" group) were examined in a separate analyses. Again, in efforts to compare probe sets best suited for use in the NormFinder analysis, in the cycle study we designated probe sets as "most representative" according to criteria described later in the current methods section and analyzed these probe sets separately as well.

All analyses from both experiments (Figure 1) are summarized as follows:

1a) HRT study - qRT-PCR: GABAergic component genes, *ALG9*, *GAPDH *and *RPL13A *expression variability compared using geNorm, NormFinder and BestKeeper algorithms.

1b) HRT study - gene microarray -general probe set selection: NormFinder relative expression stability assessments of 1444 rhesus GeneChip^® ^probe sets annotated for housekeeping genes, grouped by brain region or hormone treatment.

1c) HRT study - gene microarray - "expressed sequences" from the general probe set selection: NormFinder relative expression stability assessments of 824 probe sets showing above average signal intensity and receiving "present" calls in MAS 5.0 analysis.

1d) HRT study - gene microarray - probe set selection for popular normalizers: NormFinder relative expression stability assessments of all rhesus GeneChip^® ^probe sets annotated for 15 "popular normalizers", grouped by brain region or hormone treatment.

2a) Cycle study - gene microarray - general probe set selection: NormFinder relative expression stability assessments of 1433 human GeneChip^® ^probe sets annotated for housekeeping genes, grouped by brain region or cycle phase.

2b) Cycle study - gene microarray - most representative probe set selection: NormFinder relative expression stability assessments of 544 human GeneChip^® ^probe sets selected for the highest annotation quality among the 1433 probe sets annotated for housekeeping genes, grouped by brain region or cycle phase.

2c) Cycle study - gene microarray - popular normalizer probe set selection from among the most representative probe sets: NormFinder relative expression stability assessments of most representative human GeneChip^® ^probe sets annotated for 15 "popular normalizers", grouped by brain region or cycle phase.

### Animals

Adult (age range 9-12 years) female rhesus macaques (*Macaca mulatta*) were cared for in accordance with IACUC regulations and the National Research Council's *Guide for the Care and Use of Laboratory Animals *by the Division of Animal Resources at the Oregon National Primate Research Center (ONPRC). All procedures were ethically and legally approved by these overseeing bodies. Twelve animals were used per study, and were housed in a temperature-controlled environment where Primate Chow (Purina Mills Inc., St. Louis, MO) was made available to the animals twice daily, at 8:00 h and again at 15:00 h. This diet was supplemented with fresh fruit and vegetables, and the animals had continuous access to drinking water.

The animals were euthanized using sodium pentobarbital (25-30 mg/kg i.v.) and exsanguinated following procedures recommended by the American Veterinary Medical Association's Panel on Euthanasia. In all cases, necropsies were performed within a narrow window of time (1000-1300 h). Various postmortem tissues were collected and made available to other investigators through the ONPRC Tissue Distribution Program. After a transcardial flush with 1 liter of 0.9% saline, the brains were immediately removed, cut into blocks, and preserved in RNA*later*^® ^(Ambion, Austin, TX). Areas of interest (MBH, HPC and AMD) were subsequently dissected from tissue blocks, resulting in a total of 36 individual samples per study.

As previously described [26], all animals in the HRT study were ovariectomized (OVX). Untreated ovariectomized animals served as controls (OXV), whereas others received subcutaneous SILASTIC implants containing either 17β-estradiol (E_2_), or E_2 _and progesterone (E_2_+P_4_).

In the cycle study, daily menstrual records were established for each female based on close inspection of the monkey's perineum and cage pan for signs of menstrual bleeding. All females were determined to have regular monthly cycles of approximately 28 days. Only data obtained from healthy monkeys, as determined by the ONPRC veterinary staff, were included in the statistical analyses. Serum E_2 _levels and serum P_4 _levels were analyzed to assist in establishing an animal's menstrual cycle phase. Using menstrual cycle monitoring, tissue was timed for collection during the early follicular stage ("EF" = low E_2 _and P_4_), late follicular ("LF" = declining E_2 _after ovulation, low P_4_) and mid luteal ("ML" = low E_2 _high P_4_). Four animals were identified for each of the three phases of the cycle (total = 12). Average values for EF were E_2 _= 64.8 pg/ml, P_4 _= 0.18 ng/ml; for LF, E_2 _= 71 pg/ml, P_4 _= 0.52 ng/ml; for ML, E_2 _= 39 pg/ml, P_4 _= 4.92 ng/ml. At necropsy, menstrual stage was also verified using histological examination of the endometrium, with EF = thin endometrium, ciliated oviduct; LF = thin, proliferative endometrium with ciliated and fully secretory oviduct; ML = thickened endometrium.

### qRT-PCR

Quantitative real-time RT-PCR (qRT-PCR) and data collection were conducted using the 7900HT Fast Real-Time PCR thermal cycler and sequence detection systems (Software version 2.2.1) from Applied Biosystems (Foster City, CA). No-template (negative) controls were included for each gene analysis. Four-point standard curves were constructed using cDNA pooled equally from all brain regions and all animals. The curves were constructed from serial 5- fold dilutions and covered a cDNA dilution range of 0.2 to 0.0016 (larger fold dilutions produced unacceptably high cycle times for some genes in the study).

qRT-PCR reactions for unknowns were conducted in sealed 384-well optical plates in a total volume of 5 μl per well, using 1.0 μl of 1:5-diluted cDNA, and final concentrations of 0.25 μM Taqman^® ^TAMRA probe and 0.3 μM each of forward and reverse primers. Reactions were conducted using thermal cycler conditions of: 2 min at 50°C, 10 min at 95°C and 50 cycles of 15 s at 95°C (DNA melting) and 1 min at 60°C (primer annealing/extension). Baseline and threshold levels for amplification plots were determined automatically using the ABI sequence detection systems software version 2.2.1.

We examined expression of prospective internal reference genes (*ALG9*, *GAPDH *and *RPL13A*), as well as genes encoding GABA receptor subunits (*GABRA1*, *GABRA4*, *GABRG2*, *GABRD *and *GABRE*) and a GABA-synthesizing enzyme (*GAD1*). We compared expression stability across three conditions of ovarian steroid exposure in the three brain regions examined (MBH, HPC and AMD). These comparisons were initially made in an investigation of hormone effects on expression of GABAergic components [26], but we describe the qRT-PCR results in more detail here for further comparison against results from gene microarray analyses.

### qRT-PCR primers and probes

Primers and probes for prospective internal reference genes *ALG9*, *GAPDH *and *RPL13A *as well as GABA subunit receptors *GABRA1*, *GABRA4*, *GABRG2*, *GABRD *and *GABRE *and the enzyme *GAD1 *were designed using Primer Express 2.0 software (Applied Biosystems, Foster City, CA) from sequence sources listed in Table 1. Rhesus macaque sequences for *GAPDH *and *RPL13A *were confirmed in-house using RT-PCR primers against source sequences to amplify rhesus macaque hippocampal cDNA pooled from several individuals. These PCR products were sequenced using an ABI 3730×l DNA sequencer (Applied Biosystems) according to manufacturer's instructions.

### 1a. qRT-PCR GeNorm analysis

Quantity values calculated using the standard curve method [50] were entered into the geNorm version 3.5 VBA applet for Windows [30] and used to calculate the gene-stability measure (M) defined as the average pairwise variation of a given gene with all other considered genes. The least stable gene was then eliminated and stability based on pairwise comparisons recalculated to produce a new ranking. This sequential elimination process [34] was continued until no stability differences were detected between genes. Because one of the MBH OVX samples introduced a high level of variation in gene expression, the analysis was repeated with this sample omitted, in order to test the effects of variation in this group on relative gene expression stability output.

### 1a. qRT-PCR NormFinder analysis

Quantity values calculated using the standard curve method [50] were evaluated using the model-based variance estimation approach facilitated by the "NormFinder" Visual Basic application [31] for Microsoft Excel, available from the Molecular Diagnostics Laboratory (Aarhus University, Denmark). Samples were grouped according to brain region and stability was estimated with and without group identifiers. For each of the three brain regions, intra-group and inter-group variation [37] was calculated for each gene. Three rounds of analysis were conducted as follows: 1) All nine genes were included in order to test the stability of GABA receptor subunit genes; 2) The five most stable genes (*ALG9*, *GAPDH*, *RPL13A GAD1 *and *GABRA4*) were analyzed in attempts to maximize the quality of variation estimation methodology based on sample size; 3) Only the three predicted normalizers, *ALG9*, *GAPDH *and *RPL13A*, were analyzed to maximize estimation quality based on removal of systematic variation [37]. As described for the geNorm analysis, all tests were repeated with and without the single MBH OVX sample responsible for most of the MBH variation omitted. High gene expression variation in the MBH also prompted additional stability analysis where the MBH was considered separately from the HPC and AMD.

### 1a. qRT-PCR BestKeeper analysis

Crossing point values [38] were examined using the BestKeeper [32]Excel-based tool [51]. Standard curves were used to calculate amplification efficiency (E_A_) according to the convention E_A _= 10^(-1/slope)^. All samples were analyzed together, and in light of NormFinder analysis results highlighting GAPDH expression variation in the MBH, the MBH was analyzed separately from the AMD and HPC.

### Gene microarrays

RNA extraction and preparation of cDNA from MBH, HPC and AMD was conducted as previously described [26,52]. Both RMA and Affymetrix^® ^Microarray Suite version 5.0 (MAS 5.0) [39] analyses were conducted using methods previously described [26,52]. RMA-normalized results were analyzed using the NormFinder Visual Basic application [31] where identifiers were used to conduct two analyses (according to grouping) in each study. In the HRT study, expression values were grouped according to 1) brain region and 2) hormone treatment. For the cycle study, expression values were grouped according to 1) brain region and 2) to phase of the menstrual cycle.

### 1b. Microarray general probe set selection from the rhesus GeneChip^®^

A publicly available list of 575 human genes expressed under all conditions tested, and derived from publicly available microarray results, http://www.compugen.co.il/supp_info/Housekeeping_genes.html,

was used to identify genes with high likelihood of meeting criteria required to be considered as appropriate housekeeping genes [33]. NetAffx build number 28 (March 11, 2009) for the Affymetrix^® ^GeneChip^® ^rhesus Macaque Genome Array was used to select rhesus macaque probe sets with RefSeq mRNA transcript IDs matching genes from the publicly available list of 575 proposed human housekeeping genes [33]. Where no matches were found, probe sets were selected if gene symbols for housekeeping genes of interest were included in their annotation. If RefSeq mRNA transcript and gene symbol annotations for probe sets could not be found, the annotation build was searched by gene name.

### 1c. Microarray selection of "expressed" probe sets from the rhesus GeneChip^®^

Because many of the sequences obtained from the general probe set selection were poorly annotated or showed low expression, a second pair of NormFinder relative expression stability assessments, grouped by brain region or hormone treatment, was made using only probe sets with average expression levels meeting detection criteria using MAS 5.0 analysis. Probe sets meeting "expressed" criteria (824 probe sets in total) showed average signal detection of 200 (global scaling target intensity) or higher, and had present calls [43] in more than 50% of the animals.

### 2a. Microarray general probe set selection from the human GeneChip^®^

Using annotations from HG-U133 Plus 2.0 NetAffx build number 28 (March 11, 2009), RefSeq record numbers for mature mRNA transcripts, indicated by the prefix "NM_"[53], from the housekeeping gene list were used to identify probe sets on the Affymetrix^® ^U133 Plus 2.0 GeneChip^® ^[54] representative of the genes in question.

### 2b. Microarray most representative probe set selection from the Human GeneChip^®^

Probe set selection using this approach was designed to maximize accuracy while minimizing redundancy in the data used to compare gene expression stability. To maximize annotation consistency between the probe set sequences and the genes listed in the public database of 575 housekeeping genes, probe sets with representative public IDs matching those in the public database list were prioritized. However, if no public ID match was found, RefSeq Transcript ID listings were used. If multiple transcript IDs were listed, or multiple probe sets had the same transcript ID, where possible, we eliminated probe sets with "x" and "s" suffixes [55] where probes in the probe set may have matched transcripts from different genes. In general we selected final probe sets with the highest annotation grade (A), however, to ensure consistency with original mRNA record numbers, probe sets with B-grade annotations were selected in rare instances. Where probe sets appeared equivalent, we selected the set with the most complete mRNA coding sequence (cds), or more recent submission. If complete cds sequences overlapped then sets with the embedded sequence were selected. Where annotation was unclear, the gene was not included.

### 1d and 2c. Microarray probe sets from "popular normalizers"

Thirteen genes from the publicly available list of housekeeping genes were designated "in popular use as reference in real-time PCR" http://www.compugen.co.il/supp_info/Housekeeping_genes.html. These 13 genes together with *ALG9 *and *RPL13A *are referred to as the "popular normalizers" in this manuscript. Because the relative expression stability values calculated by the NormFinder algorithm can be argued to be more accurate when more stably expressed genes are used, we conducted separate analyses using only the popular normalizers for comparison with the larger publicly available group. Probe sets used in analyses of the popular normalizers were selected using the "general" and "representative" methods described above.

## Abbreviations

*ACTB: *gene encoding actin, beta; *ACTG1: *gene encoding actin, gamma 1; *ACVRL1: *gene encoding activin A receptor type II-like 1; *AHSA1*: gene encoding activator of heat shock 90 kDa protein ATPase homolog 1; *AKR1A1*: gene encoding aldo-keto reductase family 1, member A1; *ALDOA: *gene encoding aldolase A, fructose-bisphosphate; *ALG9: *gene encoding asparagine-linked glycosylation 9 homolog; AMD: amygdala; *API5: *gene encoding apoptosis inhibitor 5; *ARHGDIA: *gene encoding Rho GDP dissociation inhibitor (GDI) alpha; *ARHGEF7: *gene encoding Rho guanine nucleotide exchange factor (GEF) 7; *ARL2*: gene encoding ADP-ribosylation factor-like 2; *ATF4: *gene encoding activating transcription factor 4 (tax-responsive enhancer element B67); *ATP5S: *gene encoding ATP synthase, H+ transporting, mitochondrial F0 complex, subunit s (factor B); *ATP6V1F: *gene encoding ATPase, H+ transporting, lysosomal 14 kDa, V1 subunit F; *B4GALT3*: gene encoding UDP-Gal:betaGlcNAc beta 1,4- galactosyltransferase, polypeptide 3; *BRI3*: gene encoding Brain protein I3; *CALM1/CALM2/CALM3: *gene encoding calmodulin 1/2/3 (phosphorylase kinase, delta); CCND3: gene encoding cyclin D3; *CLTA: *gene encoding clathrin, light chain (Lca); *COL6A1: *gene encoding collagen alpha-1(VI) chain; *COPS6: *gene encoding COP9 constitutive photomorphogenic homolog subunit 6 (Arabidopsis); *COX4I1*: gene encoding cytochrome c oxidase subunit IV isoform 1; *COX7A2L: *gene encoding polypeptide 2-like cytochrome c oxidase subunit VIIa; *COX7C*: gene encoding cytochrome c oxidase subunit VIIc; CP: crossing point; *CSNK2B/LY6G5B: *gene encoding casein kinase 2, beta polypeptide/lymphocyte antigen 6 complex, locus G5B; Ct: cycle threshold; *CYB5R1*: gene encoding cytochrome b5 reductase 1; *DIAPH1: *gene encoding diaphanous homolog 1 (Drosophila); E_2_: 17β-estradiol; E_2_+P_4_: 17β-estradiol + progesterone; E_A_: PCR Amplification efficiency; *EEF1A1: *gene encoding eukaryotic translation elongation factor 1 alpha 1; *EEF2: *gene encoding eukaryotic translation elongation factor 2; EF: early follicular (menstrual cycle phase); *EIF1: *gene encoding eukaryotic translation initiation factor 1; *EIF4A2*: gene encoding eukaryotic translation initiation factor 4A, isoform 2 e encoding protein; GABA: gamma-aminobutyric acid; *GABRA1: *gene encoding GABAA receptor subunit alpha 1; *GABRA4: *gene encoding GABAA receptor subunit alpha 4; *GABRD: *gene encoding GABAA receptor subunit delta; *GABRE: *gene encoding GABAA receptor subunit epsilon; *GABRG2: *gene encoding GABAA receptor subunit gamma 2; *GAD1: *gene encoding glutamic acid decarboxylase 1 (67 kDa); *GAPDH: *gene encoding glyceraldehyde 3-phosphate dehydrogenase; *GAPDH: *gene encoding Glyceraldehyde-3-phosphate dehydrogenase; *GP2: *gene encoding glycoprotein 2 (zymogen granule membrane); *GPAA1: *gene encoding glycosylphosphatidylinositol anchor attachment protein 1; *GPX4: *gene encoding glutathione peroxidase 4 (phospholipid hydroperoxidase); *GRIK5: *gene encoding glutamate receptor, ionotropic kainate 5; *H6PD: *gene encoding hexose-6-phosphate dehydrogenase (glucose 1-dehydrogenase); *HAX1: *gene encoding HCLS1 associated protein X-1; *HINT1: *gene encoding histidine triad nucleotide binding protein 1; *HIST1H2BC: *gene encoding histone H2B 291B; HPC: hippocampus; *HPCAL1: *gene encoding hippocalcin-like protein 1; *HSPCB: *gene encoding heat shock protein 90kDa alpha (cytosolic), class B member 1; *INPP5K: *gene encoding inositol polyphosphate-5-phosphatase K; *LDHA: *gene encoding lactate dehydrogenase A; *LDHB: *gene encoding lactate dehydrogenase B; LF: late follicular (menstrual cycle phase); *MAPKAPK2: *gene encoding mitogen-activated protein kinase-activated protein kinase 2; MBH: arcuate nucleus of the medial basal hypothalamus; ML: mid luteal (menstrual cycle phase); *MVK: *gene encoding mevalonate kinase; *NACAP1: *gene encoding nascent-polypeptide-associated complex alpha polypeptide pseudo gene 1; *NDUFA2: *gene encoding NADH dehydrogenase (ubiquinone) 1 alpha subcomplex, 2, 8kDa; *NDUFS5: *gene encoding NADH dehydrogenase (ubiquinone) Fe-S protein 5, 15kDa (NADH-coenzyme Q reductase); *NONO: *gene encoding non-POU domain containing, octamer-binding protein; *NUDT3*: gene encoding nudix (nucleoside diphosphate linked moiety X)-type motif 3; OVX: ovariectomized; P_4_: progesterone; *PFDN1: *gene encoding prefoldin subunit 1; *PFDN5: *gene encoding prefoldin subunit 5; *PGK1: *gene encoding phosphoglycerate kinase 1; *POLR2A: *gene encoding polymerase (RNA) II (DNA directed) polypeptide A, 220kDa; *PPP2CB: *gene encoding protein phosphatase 2 (formerly 2A), catalytic subunit, beta isoform; *PRPF8*: gene encoding PRP8 pre-mRNA processing factor 8 homolog (S. cerevisiae); *PSMD2: *gene encoding proteasome (prosome, macropain) 26S subunit, non-ATPase, 2; qRT-PCR: quantitative real-time polymerase chain reaction; *RAC1: *gene encoding ras-related C3 botulinum toxin substrate 1; *RAD23A*: gene encoding RAD23 homolog A (S. cerevisiae); *RAD9A: *gene encoding RAD9 homolog; *RPL10: *gene encoding ribosomal protein L10; *RPL11: *gene encoding ribosomal protein L11; *RPL13A: *gene encoding ribosomal protein L13a; *RPL19: *gene encoding ribosomal protein L19; *RPL32: *gene encoding ribosomal protein L32; *RPL37: *gene encoding ribosomal protein L37; *RPLP1: *gene encoding ribosomal protein, large, P1; *RPS14: *gene encoding ribosomal protein S14; *RPS18: *gene encoding ribosomal protein S18; *RPS24: *gene encoding ribosomal protein S24; *RPS25: *gene encoding 40S ribosomal protein S25; *RPS27A/UBB/UBC: *gene encoding ribosomal protein S27a/ubiquitin B/ubiquitin C; *RPS27A: *gene encoding ribosomal protein S27a; *RRAGA*: gene encoding Ras-related GTP binding A; *RUVBL2*: gene encoding RuvB-like 2 protein (E. coli) *SAFB: *gene encoding Hsp27 ERE-TATA-binding protein (HET); *SARS: *gene encoding seryl-tRNA synthetase; *SDC3: *gene encoding similar to Syndecan-3 (SYND3); *SNRPA*: gene encoding small nuclear ribonucleoprotein polypeptide A; *SNX3: *gene encoding sorting nexin 3; *SPAG7: *gene encoding sperm associated antigen 7; *SSR2: *gene encoding signal sequence receptor, beta (translocon-associated protein beta); *STK24: *gene encoding serine/threonine kinase 24; *SUI1: *gene encoding putative translation initiation factor *TADA3L: *gene encoding transcriptional adaptor 3 (NGG1 homolog, yeast)-like; *TCF25: *gene encoding transcription factor 25 (basic helix-loop-helix); *TCFL1*: gene encoding vacuolar protein sorting 72 homolog (S. cerevisiae); *TMED9*: gene encoding transmembrane emp24 protein transport domain containing 9; *TTC1: *gene encoding tetratricopeptide repeat domain 1; *UBB*: gene encoding ubiquitin B; *UBC*: gene encoding ubiquitin C; *UQCRH: *gene encoding ubiquinol-cytochrome c reductase hinge protein; UQCRQ: gene encoding ubiquinol-cytochrome c reductase, complex III subunit VII, 9.5kDa; *VEGFB: *gene encoding vascular endothelial growth factor B; *VPS72*: gene encoding vacuolar protein sorting 72 homolog (S. cerevisiae); *YWHAH: *gene encoding 14-3-3n protein; *ZNF592: *gene encoding zinc finger protein 592.

## Competing interests

The authors declare that they have no competing interests.

## Authors' contributions

SGK and HFU designed and conducted animal treatment regimes, collected and processed brain tissues. NCN performed real-time RT-PCR, conducted geNorm, NormFinder and BestKeeper analyses and wrote the manuscript. All authors read, corrected and approved the final manuscript.
